# Effectiveness of Covid-19 vaccines (Covishield^TM^ and Covaxin ^®^) in healthcare workers in Mumbai, India: A retrospective cohort analysis

**DOI:** 10.1371/journal.pone.0276759

**Published:** 2022-10-27

**Authors:** Aashish Contractor, Shashikala Shivaprakash, Anjali Tiwari, Maninder Singh Setia, Tarang Gianchandani

**Affiliations:** Sir HN Reliance Foundation Hospital and Research Centre, Mumbai, Maharashtra, India; Translational Health Science & Technology Institute, INDIA

## Abstract

**Background:**

India started its vaccination programme for Coronavirus-19 infection (COVID-19) on 16 January 2021 with Covishield^TM^ (Oxford/Astra Zeneca vaccine manufactured by Serum Institute of India) and Covaxin ^®^ (Bharat Biotech, India). We designed the present study to study the effectiveness of vaccines for COVID-19 in prevention of breakthrough infections and severe symptomatic cases among health care workers in a real-life scenario in Mumbai, India. Furthermore, we also wanted to study the factors associated with this effectiveness.

**Methods:**

This is cohort analysis of secondary data of 2762 individuals working in a tertiary health care setting in Mumbai, India (16 January 2021 to 16 October 2021). Vaccination records of all groups of health care staff (including the date of vaccination, type of vaccine taken, and date of positivity for COVID-19) were maintained at the hospital. The staff were tested for COVID-19 at least once a week and when symptomatic. The observation time for everyone was divided into unvaccinated, partially vaccinated (14 days after the first dose); and fully vaccinated (14 days after the second dose). If the individual was found to be positive, the day of positivity was considered the ‘day of the event’ for that individual. We combined unvaccinated/partially vaccinated into one group and completely vaccinated in the other group. We estimated hazard ratios (HR) and their 95% confidence intervals. The vaccine effectiveness (VE) was assessed as (1-HR)*100.

**Results:**

The mean age (SD) of the study participants was 32.3 (8.3) years; majority of these individuals had taken Covishield ^TM^ (99.0%) and only 0.9% (n = 27) had taken Covaxin ^®^. The incidence rate in the overall population was 0.067/100 person-days (PD). The incidence rate was significantly higher in the unvaccinated/partially vaccinated group compared with the fully vaccinated group (0.0989 / 100 PD vs 0.0403/100 PD; p < 0.001). The adjusted HR (aHR) in the fully vaccinated group compared with the unvaccinated/partially vaccinated group in the complete cohort was 0.30 (95% CI: 0.23, 0.39). Thus, the vaccine effectiveness (VE) for full vaccination was 70% (95% CI: 61%, 77%). It remained the same in the Covishield ^TM^ only cohort. The VE in completely vaccinated and with a history of previous infection was 88% (95% CI: 80%, 93%). Only 11 health care workers required hospitalization over the entire observation period; the incidence rate in our cohort was 0.0016 / 100 PD. None of the HCWs reported any severe adverse events after vaccination.

**Conclusions:**

In this real-world scenario, we did find that complete vaccination reduced the rate of infection, particularly severe infection in health care personnel even during the severe delta wave in the country. Even among those infected, the hospitalisation rates were very low, and none died. We did not record any major side effects of vaccination in these personnel. Previous infection with COVID-19 and complete vaccination had a significantly higher effectiveness in prevention of infection.

## Introduction

On the 30 January 2020, a public health emergency was declared by the World Health Organization (WHO) following extensive laboratory tests that led to the identification of a novel coronavirus, SARS-CoV-2, as the causative agent of pneumonia in Wuhan, China [1]. The virus can spread from person-to-person via direct transmission of respiratory droplets, or indirectly via contact with contaminated surfaces [2]. A global pandemic was declared in March 2020, leading to extreme measures to control the spread of coronavirus disease 2019 (COVID-19) [3], which in turn has had a negative effect on global economies, medical infrastructures, and mental health [4]. This has increased the need to understand the kinetics of the immune response to COVID-19. As of this report, the coronavirus had multiple waves of infections in various countries; the total number of cases globally reported have been 520,668,473 with a total number of 6,262,449 deaths [5]. India has reported a cumulative number of 43,116,254 cases and 524,190 deaths till date [6].

Vaccines for COVID-19 infection were developed relatively faster as compared with earlier pandemics; in fact, by the end of 2020, countries had already started administering the doses [7, 8]. There are various types of vaccines: inactivated virus vaccine (Sinovac Biotech and Bharat Biotech); protein based vaccines (Novavax, GlaxoSmithKline); viral vector vaccines—some of the adenovirus vector vaccines are University of Oxford/Astra Zeneca [UK] (also known as Covishield ^TM^ in India manufactured by Serum Institute of India), Sputnik V (Gamaleya National Center for Epidemiology and Microbiology, Russia); mRNA vaccines (BioNTech/Pfizer [Germany, USA] and Moderna [USA]); and DNA based vaccines (ZyCoV-D vaccine [Cadila Healthcare]) [8–11]. India started its vaccination programme on 16 January 2021. The two main vaccines used initially Covishield^TM^ (Oxford/Astra Zeneca vaccine manufactured by Serum Institute of India) and Covaxin^®^ (Bharat Biotech, India) [12–14]. The vaccines were initially used for vaccinating health care workers, followed by front line workers, elderly (more than 60 years) and those with co-morbidities [15]. From May 01, 2021 onwards, the vaccination was started for all adults (≥18 years of age). Currently, apart from the above two mentioned vaccines, India has approved Sputnik V (Gamaleya National Center for Epidemiology and Microbiology, Russia), ZyCoV-D (Cadila Healthcare), Corbevax ^TM^ (Biological E. Limited, India), and Covovax ^TM^ (Serum Institute of India) for use in the population [16]. Besides the ones that are already in use, some of the other vaccines that are in various stages of approvals are: Biological E’s novel Covid-19 vaccine, BBV154 Intranasal vaccine (Bharat Biotech), and mRNA based vaccine (HGC019) (Gennova Biopharmaceuticals Ltd.) [17]. As of 16 May 2022, India had administered a total of 1,912,934,803 total cumulative doses in the country [18].

The efficacy of various vaccines ranges from 51% to more than 90%, with varying doses (one or two based on the vaccine) [8]. Most countries have now initiated booster doses for those who have been completely vaccinated. However, there was hesitancy for vaccination–even among health care workers. This hesitancy was associated with concerns about vaccine efficacy and associated side effects, and lack of trust in public health authorities [19]. Jain and co-workers reported that concerns about safety was reported by 82% and issues with vaccine efficacy was reported by 28% of medical students hesitant to take COVID-19 vaccine [19]. Thus, we designed the present study to study the effectiveness of complete vaccination (defined as two doses–according to the existing guidelines in India) for COVID-19 in prevention of breakthrough infections and severe symptomatic cases among health care workers in a real-life scenario in Mumbai, India. Furthermore, we also wanted to study the factors associated with this effectiveness.

## Methods

The present study is a cohort analysis of secondary data of 2762 individuals working in a tertiary health care setting in Mumbai, India (16 January 2021 to 16 October 2021).

### Study site and participants

Data from staff working in private tertiary health care centre in Mumbai (India) were abstracted from administrative and clinical records. Vaccination records of all groups of health care staff (including the date of vaccination, type of vaccine taken, and date of positivity for COVID-19) were maintained at the hospital. As a part of the hospital protocol, the health care staff were tested by two tests: rapid antigen testing and the reverse transcription–polymerase chain reaction (RT-PCR). The frequency of the testing was defined by the nature of their role in the hospital, and their location. However, each staff was tested at least once a week, using the RT-PCR method. They were also encouraged to test for IgG antibodies, prior to their second vaccine dose, and 14 days, post their second dose. COVID-19 infected workers were offered healthcare assistance, including medical consultations, and medications by the hospital.

We only included those individuals for whom we had information on the vaccination status and details of positivity for the entire duration of the study period. Thus, data from 2762 health workers were analysed for the present study. Data collection started from 16 January 2021 (beginning of vaccination in India) and the date of the last observation was 16 October 2021. The main outcome was positivity for COVD-19 by RT-PCR.

As stated earlier, our main objective was to assess the effectiveness of complete vaccination for COVID-19 in prevention of breakthrough infections and severe symptomatic cases among health care workers in a real-life scenario. In India, complete vaccination during this time period was defined as two doses of Covishield^TM^ or Covaxin^®^. Thus, for this analysis, we combined unvaccinated and partially vaccinated as one group. If the individual was found to be positive, the day of positivity was considered the ‘day of the event’ for that individual, and all observations after this day were not included for the current analysis.

Health care workers who tested positive after 14 days of receipt of the first dose and up to 13 days after the second dose were considered as ‘positive after first dose of the vaccine (partial vaccination)’. Health care workers who tested positive after 14 days of the second dose were considered as ‘positive after second dose of the vaccine (complete vaccination)’. Health care workers who tested positive before the first dose was considered ‘positive before the first dose’

### Specimen collection and processing

Beginning in the fall of 2020, all employees and health care workers of our hospital were tested at least once weekly with a RT-PCR test. Nasopharyngeal and orpharyngeal swabs were collected in viral transport media (VTM, HiMedia, Mumbai, India). These were collected by a trained health care worker, packed, and transported to the laboratory in the chiller box according to the protocol and guidelines suggested by the Indian Council for Medical Research. Apart from tests for diagnosis for COVID-19, health care workers who opted for antibody level testing, were tested for IgG assays. Roche Elecsys ^®^ Anti-SARS-CoV-2 S (Roche Diagnostics, F.Hoffman-La Roche Ltd. Switzerland) electrochemiluminescence immunoassay (ECLIA) for the in vitro quantitative determination of antibodies (including IgG) against spike RBD of SARS-CoV-2 in human serum was performed on Roche cobas ^®^ e 601 modules (Roche Diagnostics, F.Hoffman-La Roche Ltd. Switzerland). According to the manufacturer, the correlation test between Roche Elecsys ^®^ Anti—SARS-CoV-2 S units per mL and WHO International Standards for anti-SARS-CoV-2 immunoglobulins showed an excellent correlation (r2 = 0.9992, slope = 0.972, intercept = 0.0072), thus allowing to consider specific Roche Elecsys ^®^ Anti-SARS-CoV-2 S U/mL units equivalent to WHO International Standard BAU/mL (Binding Arbitrary Units per mL). Measuring range spanned from 0.4 BAU/mL to 2500.0 BAU/mL; values higher than 0.8 BAU/mL were considered positive.

### Statistical methods

The observation time for each individual was divided as follows: Unvaccinated (when the individual had not received any COVID-19 vaccine); partially vaccinated (14 days after the first dose); and fully vaccinated (14 days after the second dose). If the individual was found to be positive, the day of positivity was considered the ‘day of the event’ for that individual, and all observations after this day were not included for the current analysis.

As indicated earlier, we combined the unvaccinated and partially vaccinated in the same group. Initially, we estimated the proportion of new COVID-19 infection according to various demographic and clinical characteristics. Subsequently, we the data were arranged for survival analysis. We estimated the incidence rate (and its 95% confidence intervals) for COVID-19 infection according to vaccination status. We then estimated the hazard ratio (HR) and its 95% confidence interval. We chose the model based on the distribution and fit of the residuals, and the Akaike Information Criteria (AIC) and Bayesian Information Criteria (BIC). The models were built in the following sequence: null model, model with just one variable (vaccination status), and multivariate models. For the multivariate analysis, we included age, gender, and type of job profile (patient facing/not patient facing) as covariates in the models. We used the Weibull distribution for analysis of the data (based on the above-mentioned criteria). The effectiveness of the vaccine was estimated by using the formula (1-HR)*100 [20]. We did the same analysis for ‘hospitalisation due to COVID-19 infection’ as another outcome for this cohort.

### Subgroup analysis

This was a real scenario analysis. Thus, the health care professionals had been vaccinated by both the vaccines approved in India (Covishield ^TM^ and Covaxin ^®^). Since most of them had received the former, we did a sub-group analysis of patients who had received the Covishield ^TM^ vaccine. We also categorised the patients based on history of previous COVID-19 infection (prior to 16 January 2021) and current vaccination status in these four groups: 1) unvaccinated/partial vaccinated and no history of previous infection; 2) unvaccinated/partial vaccinated and history of previous infection; 3) complete vaccination and no history of previous infection; and 4) complete vaccination and no history of previous infection. We estimated the incidence rates and hazard ratios for these groups. As with the complete cohort analysis, we used multivariate models for this sub-group analysis. We also tested the difference in the hazard ratios using the methods described by Altman.

The study was approved the Institutional Ethics Committee (Reference No: HNH/IEC/2021/OCS dated 16 June 2021, amendment dated: 02 June 2022). Anonymised data were used for analysis and waiver of consent was granted by the Ethics Committee.

## Results

**Fig 1:**
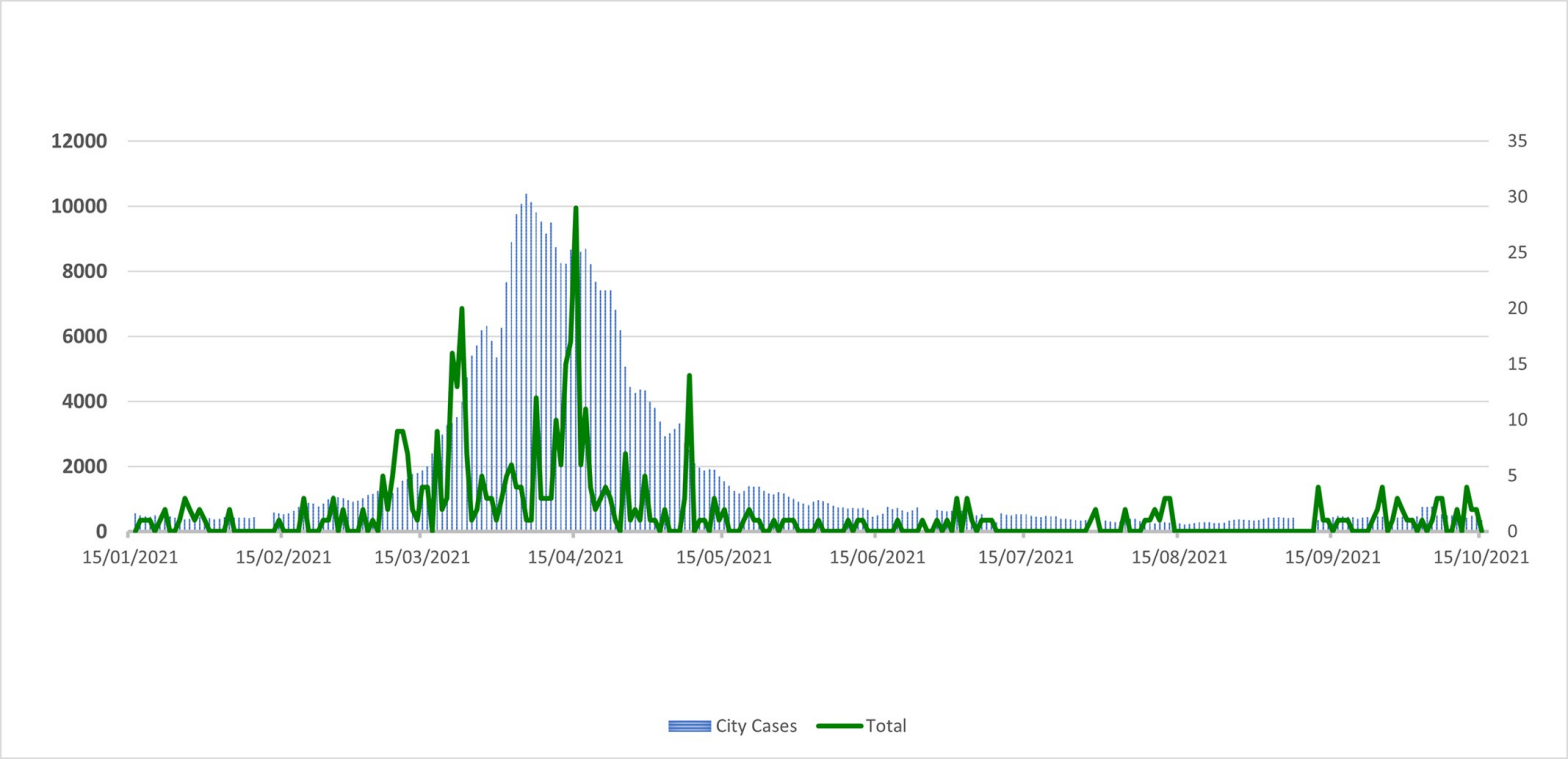
Figure showing the total number of cases (3-day moving average) in the city of Mumbai, and total number of new infections in our cohort, Mumbai, India.

The mean age (SD) of the study participants was 32.3 (8.3) years. About 45% of the participants were female and 55% were male; there were no significant differences in the incidence of COVD-19 across both the genders. In our study, 17% had a previous history of COVID-19 infection (prior to January 16, 2021); the incidence proportion of COVID-19 was significantly lower in those who had a previous history of COVID-19 compared with those who did not (3.3% vs 19.1%; p<0.001). Majority of these individuals had taken Covishield ^TM^ (99.0%) and only 0.9% (n = 27) had taken Covaxin ^®^. We have presented characteristics and incidence proportion according to these characteristics in Table 1. As seen in Fig 1, most of the COVID-19 infections in our cohort coincided with the increase in the number of the cases in the city of Mumbai. These were during late March, April, and early May 2021.

**Table 1:** Table showing the proportion of COVID-19 infections in a cohort of 2762 health care personnel in a tertiary care centre, Mumbai, India.

Characteristics	Total	COVID-19 Positive	COVID-19 Negative	P value
All	2762 (100)	451 (16.3)	2311 (83.7)	
Age (years)				
19–29	1224 (44.3)	182 (14.9)	1042 (85.1)	0.053
30–39	1041 (37.7)	196 (18.8)	845 (81.2)	
40–49	385 (13.9)	57 (14.8)	328 (85.2)	
> = 50	112 (4.1)	16 (14.3)	96 (85.7)	
Gender				
Male	1528 (55.3)	250 (16.4)	1278 (83.6)	0.96
Female	1234 (44.7)	201 (16.3)	1033 (83.7)	
Type of work				
Patient facing	1533 (55.5)	260 (16.9)	1273 (83.0)	0.32
Non-patient facing	1229 (44.5)	191 (15.5)	1038 (84.5)	
Type of vaccine				
Covishield ^TM^	2735 (99.0)	450 (16.5)	2285 (83.6)	0.11
Covaxin ^®^	27 (0.9)	1 (3.7)	26 (96.3)	
Previous history of COVID-19 infection				
Yes	481 (17.4)	16 (3.3)	465 (96.7)	<0.001
No	2281 (82.6)	435 (19.1)	1846 (80.9)	

**Fig 2:**
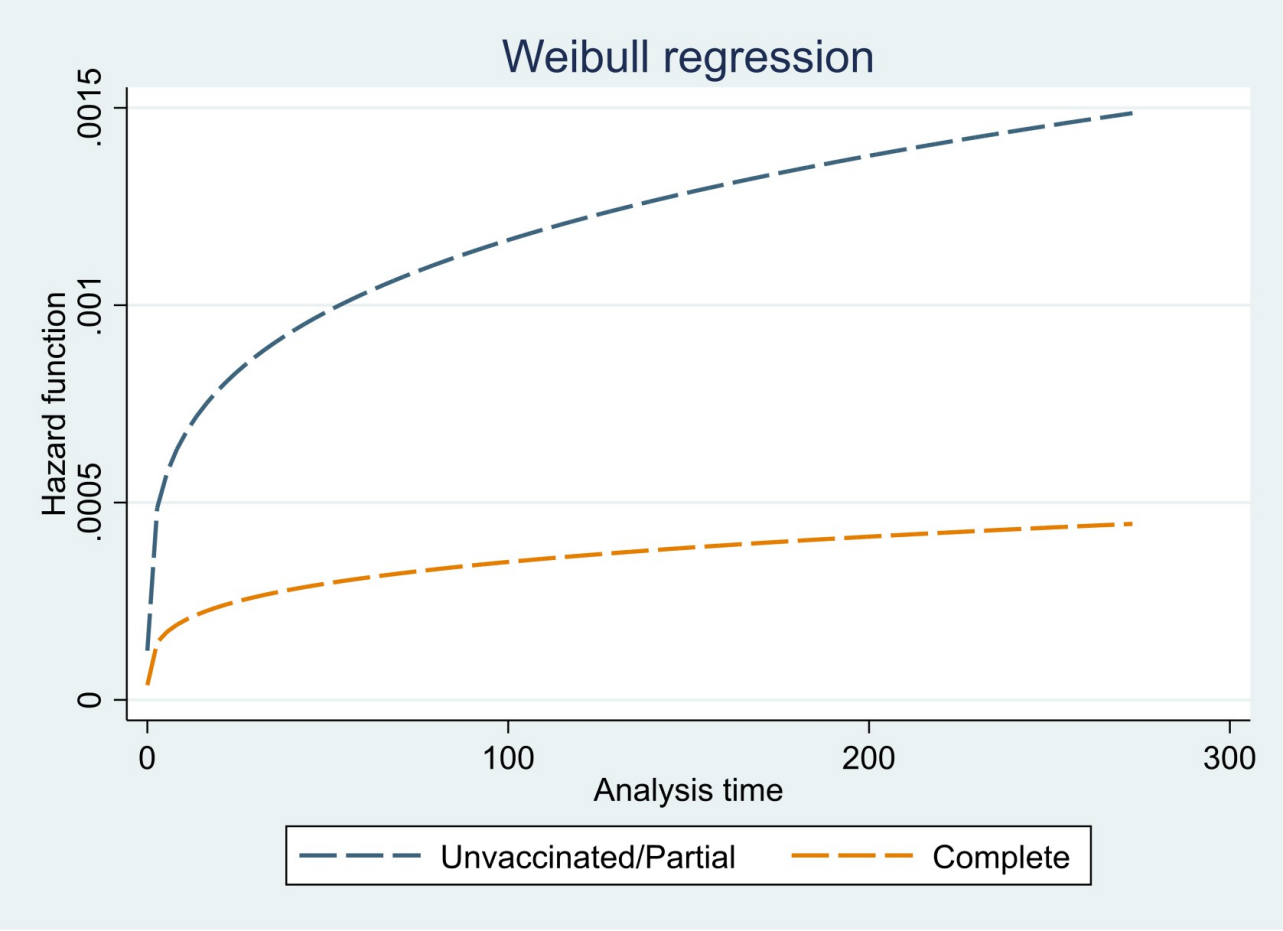
Hazard function for unvaccinated/partially vaccinated group and completely vaccinated group in 2762 health care staff in Mumbai, India.

The total observation time of these 2762 individuals was 672,147 person days (PDs). Of this, 307,405 PDs was unvaccinated/partial vaccinated time and 364,742 was fully vaccinated time. We observed 451 events (positivity) in our cohort; none of the health care workers were infected twice during the observation period. Thus, the overall incidence proportion of COVID-19 in our cohort 16.3% (95% CI: 14.9% to 17.7%). However, the incidence rate in the overall population was 0.067/100 PD. The incidence rate was significantly higher in the unvaccinated/partially vaccinated group compared with the fully vaccinated group (0.0989 / 100 PD vs 0.0403/100 PD; p < 0.001). The incidence rate was highest in the unvaccinated/partially vaccinated group and those who did not have a history of previous COVID-19 infection. We have presented the hazard function in unvaccinated/partial and completely vaccinated groups in Fig 2.

The adjusted HR (aHR) in the fully vaccinated group compared with the unvaccinated/partially vaccinated group in the complete cohort was 0.30 (95% CI: 0.23, 0.39). Thus, the vaccine effectiveness (VE) for full vaccination was 70% (95% CI: 61%, 77%). It remained the same in the Covishield ^TM^ only cohort. When we analysed the data according to history of previous COVID-19 infection and vaccination status, we found that VE was higher in those who had reported previous history of infection (prior to January 16, 2021). The VE in completely vaccinated and with a history of previous infection was 88% (95% CI: 80%, 93%). We have presented the details of person time, number of events, incidence rates, unadjusted HRs, and adjusted HRs for COVID-19 infection in Table 2. The adjusted HR (0.12, 95% CI: 0.07, 020) for completely vaccinated with previous history of infection was significantly lower compared with those who were completely vaccinated (p = 0.01).

**Table 2:** Table showing the incidence rate of COVID-19 infections and hazard ratios (with 95% confidence intervals) in a cohort of 2762 health care personnel in a tertiary care centre, Mumbai, India.

Characteristics	Total Person days	Events	Incidence (per 100 PD) (95% Confidence intervals)	Unadjusted Hazard ratios (95% Confidence intervals)	Adjusted Hazard ratios * (95% Confidence intervals)
Total Cohort	672147	451	0.067 (0.0611, 0.0735)		
Vaccination Status					
Unvaccinated/Partial	307405	304	0.0989 (0.0884, 0.111)	Reference	Reference
Complete	364742	147	0.0403 (0.0343, 0.0474)	0.30 (0.23, 0.39)	0.30 (0.23, 0.39)
Only Covishield					
Unvaccinated/Partial	303086	303	0.0999 (0.0893, 0.112)	Reference	Reference
Complete	361851	147	0.0406 (0.0345, 0.0478)	0.30 (0.23, 0.39)	0.30 (0.23, 0.39)
Previous Infection & Vaccination Status					
Unvaccinated/Partial and No Previous infection	254291	303	0.1192 (0.1065, 0.1334)	Reference	Reference
Unvaccinated/Partial and Previous infection	53114	1	0.0019 (0.0003, 0.0134	0.02 (0.01, 0.11)	0.02 (0.01, 0.11)
Complete and No Previous Infection	287703	132	0.0459 (0.0386, 0.0544)	0.29 (0.22, 0.38)	0.29 (0.22, 0.38)
Complete and Previous Infection	77039	15	0.0195 (0.0117, 0.0323)	0.13 (0.07, 0.21)	0.12 (0.07, 0.20)

* Models were adjusted for age, gender, and type of work

Only 11 health care workers required hospitalization over the entire observation period. The overall incidence of hospitalization was 0.40%, and the incidence rate in our cohort was 0.0016 / 100 PD. All these cases were in the unvaccinated/partially vaccinated group, specifically in those who did not have a prior history of COVID-19 infection (Table 3). However, none of these were serious, required admission to the ICU, and there were no deaths reported in our cohort. The median (IQR) value of IgG antibodies to N protein was 14.47 (2.12, 140.25) BAU/mL and to the S protein was 250 (246.4, 250) BAU/mL among hospitalized patients who were partially vaccinated (at least one does of the vaccine). None of the HCWs reported any severe adverse events after vaccination.

**Table 3:** Table showing the proportion of hospitalisations due to COVID-19 infection in a cohort of 2762 health care personnel in a tertiary care centre, Mumbai, India.

Characteristics	Total	Hospitalisation	Not hospitalised	P value
All	2762 (100)	11 (0.40)	2751 (99.6)	
Age (years)				
19–29	1224 (44.3)	2 (0.2)	1222 (99.8)	0.10
30–39	1041 (37.7)	5 (0.5)	1036 (99.5)	
40–49	385 (13.9)	4 (1.04)	381 (98.9)	
> = 50	112 (4.1)	0 (0.0)	112 (100.0)	
Gender				
Male	1528 (55.3)	8 (0.5)	1520 (99.5)	0.36
Female	1234 (44.7)	3 (0.2)	1231 (99.8)	
Type of work				
Patient facing	1533 (55.5)	7 (0.5)	1526 (99.5)	0.76
Non-patient facing	1229 (44.5)	4 (0.3)	1225 (99.7)	
Type of vaccine				
Covishield ^TM^	2735 (99.0)	11 (0.40)	2724 (99.6)	>0.99
Covaxin^®^	27 (0.9)	0 (0.0)	27 (100.0)	
Previous history of COVID-19 infection				
Yes	481 (17.4)	0 (0.0)	481 (100.0)	0.23
No	2281 (82.6)	11 (0.5)	2270 (99.5)	

## Discussion

Thus, in this study, we found that the overall vaccine effectiveness in the completely vaccinated health care workers (two doses) was 70% (95% confidence intervals 61% to 77%). This effectiveness increased to 88% in those who had a history of COVID-19 infection; this increase was statistically significant. Thus, previous infection with COVID-19 appeared to increase the vaccine effectiveness. Furthermore, even among the infected, the vaccine was very protective against severe form of the infection. The rate of hospitalization was very low in our cohort and no deaths were reported. The period of this study covered the duration of the second wave which was severe compared with the first wave in India.

Numerous studies have assessed the effectiveness of various corona vaccines; these have been large population-based studies as well as in relatively smaller cohorts. For instance, a large population-based study in Peru of CoronaVac vaccine which is an inactivated SARS-CoV-2 vaccine–found the vaccine effectiveness in those who were completely vaccinated individuals to be 65.9% [20]. Another large population-based study evaluated the effectiveness of four different vaccines–CoronaVac (inactivated virus), ChAdOx1 nCoV-19 and Ad26.COV2.S (viral vector type vaccine), and BNT162b2 (mRNA vaccine) [21]. They found that effectiveness of the ChAdOx1 nCoV-19 vaccine was 56.0% (95% CI: 51.4%, 60.2%); this vaccine was similar to the one used by most of the HCWs in our study. Indian studies have also provided information on the VE of ChAdOx1 nCoV-19 (Covishield ^TM^) and BBV-152 (Covaxin ^®^), the two main vaccines used in the country. The VE for the former vaccine ranged from 54% to 95% in preventing break-through infections different regions in India; this was in individuals who had been completely vaccinated [22–24]. Some other authors [25, 26] had recruited study participants who may have received either of the vaccine–as in our study. Indeed, a study in the HCWs found the effectiveness of complete vaccination to be 65% against development of infection [26]. Other studies have reported the VE of BBV-152 (Covaxin ^®^); the reported estimate varied from 29% to 78% [27, 28]. We did not have sufficient number of HCWs in the Covaxin ^®^ group, hence, we could not compare the VEs between these two types of commonly used vaccines in India. However, a recent review [29] on immunogenicity and effectiveness of these two vaccines reported that even though the VE may be slightly lower for Covaxin ^®^ compared with Covishield ^TM^, the former is more likely to be effective in situations where the structure of the spike protein alters.

As seen in our study, complete vaccination was very effective against the severe form of the infection. The hospitalisation rate was very low, and no deaths were reported in our cohort. Previous VE studies have also found complete vaccination to be highly effective against severe symptomatic infections [30–32]. For instance, a study in health care workers and frontline workers in India found the VE in prevention of deaths due to COVID-19 infection to be 98.5% [24]. Another study in police force found the VE in prevention of deaths was 95% [33]. A study done exclusively among health care workers during the second wave (delta wave) in India also found high effectiveness in prevention of severe cases (requirement of oxygen, admission to the intensive care units, and deaths) [26]. Thus, complete vaccination was useful in prevention of the severe form of COVID-19 infection even during deadly second wave of the pandemic in India.

The VE was significantly better in those who had a previous infection with COVID-19 infection. Several studies have shown that previous infection with COVID-19 infection may confer immunity against re-infection [34–37]. Systematic reviews and meta-analysis of re-infection have reported that the protection of re-infection was about 87% to 90% [38, 39]. Studies have shown the re-infection rates to be lower in those who were previously infected with SARS-CoV-2 infection and subsequently vaccinated [40]. They also reported that the duration of the protective effect in these individuals was longer. A study in HCWs found that re-infection rate in fully vaccinated individuals was 07.26 per 100 PY, and the VE was 86% [41]. Some studies, however, have reported that fully vaccinated individuals may carry the same amount of virus as unvaccinated individuals and naturally acquired immunity was more protective against infection and severity of disease in the case of Delta variant [42, 43]. Klein [44], however, suggests that though studies have found that vaccination has a better protective effect than previous infection alone, the level of boosting required needs more evidence and monitoring.

There were some limitations in our study. Though, we had values for antibodies for some HCWs, this was not a mandatory requirement of the hospital COVID-19 management protocol. Previous reviews [45, 46] have discussed the immunogenicity of these vaccines, and compared the antibody and cellular immunity response in them. They reported that the antibody response was better in Covishield ^TM^ and cellular response was better in the Covaxin ^®^ group [47, 48]. We did not assess either the humoral or the cellular response to these vaccines in our population. We combined the unvaccinated and partially vaccinated in the same group, and compared it with completed vaccinated group. Though some earlier studies have used unvaccinated, one vaccine, and two vaccines as separate groups, during the period of data collection, two doses each of Covishjeld ^TM^ and Covaxin ^®^ were considered as complete vaccination. Our main objective was to estimate the effectiveness of complete vaccination in health care workers in a real-life scenario. We did not isolate the strain of the virus. However, during the period covered by our study, the predominant variant was the delta variant–the wave was severe compared with the one in the year 2020.

Nonetheless, the study provides useful information on real-world experience of vaccination effectiveness in a relatively closed cohort of health care workers. We covered a period of 10 months. All the HCWs were examined at least once every week even if they were asymptomatic. Thus, it is less likely that we have missed any infection. In this real-world scenario, we did find that complete vaccination reduced the rate of infection, particularly severe infection in health care personnel even during the severe delta wave in the country. Even among those infected, the hospitalisation rates were very low, and none died. We did not record any major side effects of vaccination in these personnel. Previous infection with COVID-19 and complete vaccination had a significantly higher effectiveness in prevention of infection. Thus, in our study, complete vaccination appeared to be safe and effective; hence, even though there were some inhibitions, we would strongly encourage complete vaccination (at least two doses) in the population.
